# Histological findings of diabetic kidneys transplanted in non-diabetic recipients: a case series

**DOI:** 10.1007/s11255-023-03552-x

**Published:** 2023-03-20

**Authors:** Giorgia Comai, Valeria Corradetti, Claudia Bini, Francesco Tondolo, Lilio Hu, Sabrina Valente, Gianandrea Pasquinelli, Deborah Malvi, Francesco Vasuri, Matteo Ravaioli, Michele Provenzano, Gaetano La Manna

**Affiliations:** 1 Nephrology, Dialysis and Renal Transplant Unit, IRCCS - Azienda Ospedaliero-Universitaria di Bologna, Via Massarenti 9, 40138 Bologna, Italy; 2 Biotechnology and Methods in Laboratory Medicine, Department of Experimental, Diagnostic and Specialty Medicine (DIMES), Alma Mater Studiorum University of Bologna, Bologna, Italy; 3 Pathology Unit, IRCCS - Azienda Ospedaliero-Universitaria di Bologna, Via Massarenti 9, 40138 Bologna, Italy; 4 General Surgery and Transplantation Unit, IRCCS - Azienda Ospedaliero-Universitaria di Bologna, Via Mas-Sarenti 9, 40138 Bologna, Italy; 5 Alma Mater Studiorum, University of Bologna, 40138 Bologna, Italy

**Keywords:** Extended criteria donor, Diabetic donor, Diabetic nephropathy, Donor histology, Kidney transplant

## Abstract

**Background:**

Diabetic donors are recognized as a reliable source of organs, although the discard rate of kidneys is still high. Few data are available on the histological evolution of these organs especially on kidneys transplanted into non-diabetic patients who remain euglycemic.

**Methods:**

We describe the histological evolution of ten kidney biopsies performed on non-diabetic recipients of diabetic donors.

**Results:**

Mean donor age was 69 ± 7 years, 60% were males. Two donors were treated with insulin, eight with oral antidiabetic drugs. Mean recipient age was 59.9 ± 7 years, 70% were males. The pre-existing diabetic lesions identified in the pre-implantation biopsies, encompassed all histological classes, and were associated with mild IF/TA and vascular damages. The median follow-up was 59.5 [IQR 32.5–99.0] months; at follow-up, 40% of cases did not change histologic classification, two patients with class IIb downgraded to IIa or I and one with class III downgraded to IIb. Conversely, three cases showed a worsening, from class 0 to I, I to IIb or from IIa to IIb. We also observed a moderate evolution of IF/TA and vascular damages. At follow-up visit, estimated GFR was stable (50.7 mL/min vs. 54.8 at baseline) and proteinuria was mild (51.1 ± 78.6 mg/day).

**Conclusions:**

Kidneys from diabetic donors show variable evolution of the histologic features of diabetic nephropathy after transplant. This variability may be associated to recipients characteristics such as euglycemic milieu, in case of improvement, or obesity and hypertension, in case of worsening of histologic lesions.

## Introduction

Diabetic donors (DD) are recognized as a reliable source of organs. In fact, Cohen et al. demonstrated that patients that received a DD kidney had a considerable survival benefit compared with patients who remained on the waitlist [1]. Nevertheless, the long-term survival of kidneys from diabetic donors is significantly lower than that of kidneys from non-diabetic (ND) donors, although the absolute difference in graft survival is small [2]. It has been demonstrated that the negative impact of donor diabetes on graft and patient survival is dependent on the recipient diabetic status: DD kidneys transplanted into diabetic recipients have been associated with the highest risk of all-cause graft loss and patient mortality, compared to all other donor/recipient combinations in terms of diabetic status [3, 4].

The available literature is mainly focused on the functional evaluation of the graft, while scarce is the histological evaluation of the lesions from diabetes comparing the pre-implantation versus the follow-up biopsies. Khan et al. analyzed the clinical and histological outcomes of diabetic or ND kidneys transplanted in recipients with or without diabetes. They focused their attention on the presence of mild diabetic nephropathy changes in post-perfusion biopsies and on the evolution towards stable or progressive nephropathy, identified in for-indication biopsies [5, 6]. Moreover, few cases of possible reversal of diabetic lesions at the restoration of euglycemia after kidney transplant have been published [7, 8]. These studies are the first to analyze the histology in kidney transplant from DD and to suggest that normoglycemia in the recipient may be important, but unfortunately, they do not analyze the histology of those recipients that did not develop diabetes and are limited by the different and relatively short follow-up times [9, 10].

Of note, all the studies mentioned above describe a population of donors relatively young and with low Kidney Donor Profile Index (KDPI), an index routinely used in the United States for the allocation of organs, that reflects the comorbidities of the donors and has a relationship with the outcome of the kidney, the higher (> 80%) the KDPI the lower the outcome. Indeed, data on the histological evolution and clinical outcomes of kidneys from DD with comorbidities that worsen KDPI, such as age or cardiovascular diseases, are still lacking.

On our part, although in a small series of cases, this contribution is the first that addresses the histological evolution of diabetes and chronic lesions in a series of ND recipients of kidneys from diabetic donors.

## Materials and methods

The aim of our study was to evaluate the changes in diabetic histological features in kidney transplant patients that received a diabetic donor organ. We performed a retrospective analysis of electronic medical records of deceased donors enrolled from 2004 to 2015 at the Sant’Orsola Hospital–University of Bologna, Italy. Among them, we selected all those with a diagnosis of type 1 and type 2 diabetes (T2D) prior to expiration and with an available pre-implantation kidney biopsy. We identified a case series of 10 kidney transplants whose recipients, at the time of analysis, had not a history of diabetes, were still on follow-up and had executed at least one biopsy, either for cause or per protocol [3, 11]. Clinical and laboratory data were collected at the time of biopsy.

### Clinical data

Clinical information on donors was obtained through the electronic medical records provided by the Regional Transplant Center of Emilia Romagna, based in Bologna, Italy. The clinical information recorded were age, sex, body mass index (BMI), history of hypertension, cardiovascular events, diabetes and relative drugs, type of death, KDPI, cold ischemia time, creatinine, GFR calculated using the CKD-EPI formula, proteinuria determined on urine spot collections. Clinical information on recipients was collected through the medical records available at the Kidney Transplant Center of Sant’Orsola Hospital–University of Bologna, Italy. The clinical information recorded were age, sex, BMI, original nephropathy, history of hypertension, cardiovascular events, diabetes and relative drugs, HLA matches, delayed graft function (DGF), creatinine, GFR calculated using the CKD-EPI formula, proteinuria determined on 24-h collection, immunosuppressive regimens. The study was authorized by the internal Ethical Committee (185/2020/Sper/AOUBo).

### Histopathological analysis

The renal tissue was fixed in Serra solution and embedded in paraffin. Slices were cut at 3-µm thickness and stained with hematoxylin–eosin, periodic acid-Shiff and Masson’s trichrome stains. Transmission Electron Microscopy (TEM) was executed to measure the thickness of the Glomerular basement membrane (GBM). TEM was performed on specimens of renal tissue fixed in glutaraldehyde and was available for 7 patients. For the examination we used a Philips CM10 (FEI Company, Milan, Italy) TEM equipped with Gatan camera; for each sample, five digital images were randomly acquired using the FEI proprietary software Olympus SIS-Megaview-SSD digital camera. GBM thickness was measured in twelve different positions, at 13,500 of magnification. Renal tissue specimens were scored by two pathologists not in contact with each other and unaware of the patient’s clinical data. Agreement between pathologists was calculated by means of Kappa Cohen coefficient of agreement. The discordant cases were discussed collegially. Samples were scored in accordance to the established histopathological classification of diabetic nephropathy (DN) [7, 11]. The DN score has been created in 2010 and is accepted worldwide for the histological definition of DN; it consists of a six stage-score listed as follows: 0 no diabetic lesions; I = mild or nonspecific light microscopy changes and EM-proven GBM thickening; IIa = Mild mesangial expansion; IIb = Severe mesangial expansion; III = Nodular sclerosis (Kimmelstiel–Wilson lesion); IV = Advanced diabetic glomerulosclerosis [7, 11, 12]. Interstitial lesions were graded considering scores 0 to 3 on the basis of percentage of interstitial fibrosis and tubular atrophy (0 no IFTA, 1: < 25%; 2: 25–50%, 3: > 50%), according to DN and Banff classification. Similarly, vascular lesions graded the severity of arteriolar hyalinosis (0: absent, 1: at least one area, 2: more than one area) and atherosclerosis (0: No intimal thickening,1: Intimal thickening less than thickness of media, 2: Intimal thickening greater than thickness of media). As for standard practice, biopsies were scored according to Banff classification, the oldest samples were evaluated according to the last classification available [11].

## Results

### Clinical data

**Fig. 1:**
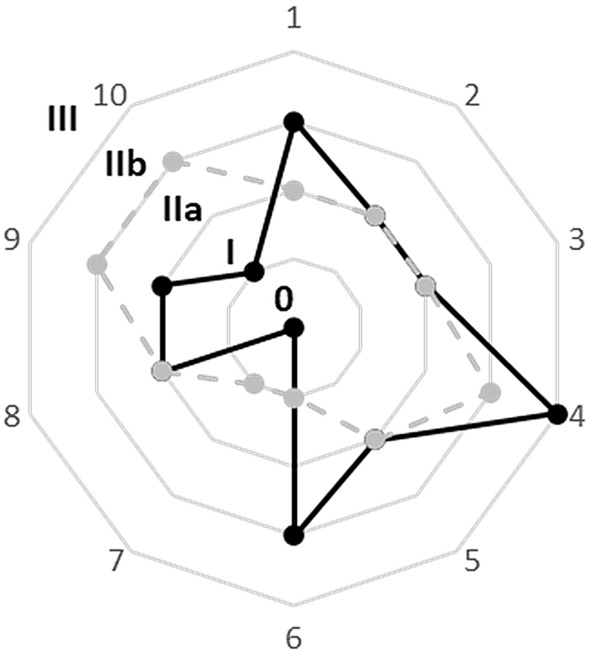
Radar plot depicting the glomerular histological scores of DN classification score (from 0 to III) for the 10 patients enrolled in the study. Black solid lines and black points represent pre-implantation biopsy scores. Grey dashed lines and grey points represent biopsy scores at follow-up visit. The ten patients are placed in the corners of the radar and the relative scores can be identified by moving from the patient number towards the center of the radar (0 value)

Among the kidney recipients from diabetic donors, we isolated 10 patients that were non-diabetic neither pre- nor post- transplantation. The characteristics of donors and recipients are summarized in Tables 1 and 2, respectively, while Fig. 1 shows the evolution over time of histological scores.

**Table 1:** Clinical and demographics characteristics of the donors

	Patient number										
Variables	1	2	3	4	5	6	7	8	9	10	Overall
Age, years	75	62	69	69	54	66	77	73	77	68	69.0 ± 7.2
Sex, M/F	M	F	M	F	M	M	M	F	F	M	F 40%
BMI, kg/m^2^	22.7	39.1	32.7	24.1	30.4	26.2	29.1	29.1	29.3	35.5	29.8 ± 5
Hypertension, yes/no	Yes	Yes	Yes	Yes	Yes	No	No	Yes	Yes	No	Y 70%
CVD, yes/no	No	Yes	Yes	No	No	Yes	Yes	No	Yes	No	50%
DM, type 1–2	2	2	2	2	2	2	2	2	2	2	100%
Insulin, yes/no	No	No	No	No	No	Yes	Yes	–	No	–	20%
Oral antidiabetic drugs,	Metformin	Metformin	Metformin	Glibenclamide, Metformin	–	No	No	–	Gliclazide	–	50%
Serum creatinine, mg/dl	1.44	0.99	0.74	0.79	0.80	1.20	0.86	0.60	0.94	0.93	0.9 ± 0.2
eGFR, ml/min/1.73m^2^	47.2	60.4	94.0	76.4	101.3	62.6	83.6	90.4	58.5	84.0	75.8 ± 18.9
Proteinuria, mg/dl	0	30	0	15	0	0	0	15	–	30	10 ± 13
DBD/DCD	DBD	DBD	DBD	DBD	DBD	DBD	DBD	DBD	DBD	DBD	100%
ECD, yes/no	Yes	Yes	Yes	Yes	Yes	Yes	Yes	Yes	Yes	Yes	100%
KDPI, %	100	99	98	99	80	89	99	100	100	93	95.7 ± 6.6
Cold ischemia time, h:min	15:00	12:15	10:30	14:25	13:00	12:50	11:00	20:25	12:30	8:30	13:19 ± 3:10

*BMI* body mass index, *CVD* cardiovascular disease, *DM* diabetes mellitus, *DBD* donor from brain death, *DCD* donor from circulatory death, *ECD* extended criteria donor, *KDPI* kidney donor profile index, *eGFR* estimated glomerular filtration rate

**Table 2:** Recipient’s characteristics at baseline and follow-up visits

	Patient number										
Variables at baseline	1	2	3	4	5	6	7	8	9	10	Overall
Age, years	63	63	59	53	47	60	67	65	69	53	59.9 ± 7
Sex, M/F	M	F	M	M	M	F	M	F	M	M	F 30%
BMI, kg/m^2^	23.3	18.4	25.2	22.4	25.1	27.7	22.4	22.9	29.2	32.1	24.9 ± 3.9
Cause of kidney disease	–	–	Anti GBM GN	ADPKD	GN	GN	ADPKD	ADPKD	IgAN	–	
Hypertension, yes/no	Yes	Yes	Yes	Yes	Yes	Yes	Yes	Yes	Yes	Yes	100%
CVD, yes/no	No	No	No	No	No	No	No	No	Yes	No	10%
DM, yes/no	No	No	No	No	No	No	No	No	No	No	100%
DGF, yes/no	Yes	No	Yes	No	No	No	No	No	No	Yes	Y 20%
DKT, yes/no	No	Yes	No	No	No	Yes	No	No	No	No	Y 20%
HLA matches, number	1	3	2	3	3	2	3	2	6	5	3 ± 1.5
Serum creatinine, mg/dl	1.44	0.99	0.74	2.20	2.30	0.90	1.40	1.20	1.30	3.30	1.6 ± 0.8
eGFR, ml/min/1.73m^2^	51.3	60.6	101.0	33.0	32.6	92.5	51.6	47.4	58.2	20.2	54.8 ± 25.5
Proteinuria, mg/die	400	984	918	–	–	400	700	205	748	196	800.4 ± 576.4
Induction therapy	ATG	ATG	ATG BAS	BAS	BAS	ATG	BAS	BAS	BAS	BAS	ATG 40%
Maintenance therapy	FK MPA ST	FK MPA ST	FK MPA ST	FK MPA ST	CsA MPA ST	FK MPA ST	FK MPA ST	FK MPA ST	CsA Eve ST	FK MPA ST	
Variables at follow-up visit											
Biopsy timing after transplant, months	27	19	12	69	49	60	59	128	114	109	64.6 ± 40.8
Protocol (P) or for cause biopsy	P	Cr rise	PTU rise	P	Cr rise	P	PTU rise	P	Cr rise	Cr rise	
Serum creatinine, mg/dl	1.06	0.74	2.78	1.25	2.87	0.96	1.67	0.92	1.81	2.79	1.7 ± 0.8
eGFR, ml/min/1.73m^2^	75.0	86.2	23.8	65.3	24.9	64.3	41.7	47.4	58.2	20.2	50.7 ± 22.9
Proteinuria, mg/die	0	150	30	20	220	20	ND	0	70	20	51.1 ± 78.6
Maintenance therapy	FK MPA ST	FK MPA ST	FK MPA ST	FK MPA ST	CsA MPA ST	FK MPA ST	FK MPA ST	FK MPA ST	CsA EVE ST	FK MPA ST	
BMI, kg/m^2^	21.5	23.6	26.2	24.4	25.4	26.8	24.2	26.3	28.2	41.3	26.6 ± 5.7
Hypertension, yes/no	Yes	Yes	Yes	Yes	Yes	Yes	Yes	Yes	Yes	Yes	100%
Anti-hypertensive drugs, number	0	1	3	1	1	3	3	2	2	3	1.9
CVD after transplant, yes/no	No	No	No	No	No	No	No	No	No	No	0%

*BMI* body mass index, *anti* *GBM* anti glomerular basement membrane, *GN* glomerulonephritis, *ADPKD* autosomal dominant polycystic kidney disease, *IgAN* IgA nephropathy, *Cr* serum creatinine, *PTU* proteinuria, *CVD* cardiovascular disease, *DKT* double kidney transplant, *HLA* human leukocyte antigens, *DGF* delayed graft function, *GFR* glomerular filtration rate, *ATG* anti thymocyte globulins, *BAS* basiliximab, *FK* tacrolimus, *MPA* mycophenolic acid, *ST* steroids, *EVE* everolimus, *CsA* ciclosporin A

The mean age of donors was 69 ± 7.2 years-old, 60% of them were male, the mean BMI was 29.8 ± 5 kg/m^2^; 70% of them were hypertense and 50% had a history of cardiovascular disease (CVD). All patients were T2D, on insulin therapy in two cases, and half of them were on oral antidiabetics. Mean serum creatinine at donation was 0.9 ± 0.2 mg/dl, eGFR 75.8 ± 18.9 ml/min/1.73 m^2^, urinary protein 10 ± 13 mg/dl, all ECD-DBD donors with a mean KDPI 95.7 ± 6.6% and mean cold ischemia time 13:19 ± 3:10 h.

Recipients aged 59.9 ± 7 years, 70% were males and the mean BMI was 24.9 ± 3.9 kg/m^2^ at baseline (Table 1). The original nephropathy was unknown in three cases, ADPKD in three cases, one had anti-GBM GN, one IgA-GN, other GN in two patients. All recipients were hypertensive, non-diabetic, both at transplant and at follow-up, and 10% of them had a history of CVD. The HLA-matches were 3 ± 1.5, induction therapy consisted in ATG (40% of cases) or Basiliximab (60% of cases); two patients received a double kidney transplant and 20% of patients presented DGF after transplant. At discharge, creatinine was 1.6 ± 0.8 mg/dl, GFR 54.8 ± 25.5 ml/min/1.73m^2^, proteinuria 800.4 ± 576.4 mg/day.

All but two patients received standard maintenance therapy with steroids, tacrolimus and mycophenolic acid; two patients were on steroids, CsA and Everolimus. Biopsies were performed per protocol in four cases and for cause in six patients. The median follow-up and timing of the biopsy was 59.5 [IQR 32.5–99.0] months; at follow-up, creatinine was 1.7 ± 0.8 mg/dl, GFR was 50.7 ± 22.9 ml/min/1.73m^2^, and proteinuria was 51.1 ± 78.6 mg/day. All patients had stayed on the same immunosuppressive regimens introduced at transplant, had not a history of rejection and the research for donor specific antibodies was negative.

### Histopathological analysis

The coefficient of agreement was high as testified by the Kappa Cohen coefficient (K = 0.85). The evaluation of the biopsies according to the Banff classification [11] showed minor changes or class V lesions (IF/TA), no diagnosis of rejection neither cellular nor humoral, C4d was negative in all cases. Calcineurin inhibitors toxicity, evaluated as the presence of nodular arteriolar hyalinosis extended to the tunica media and strip fibrosis, was not found in any patients.

- (a) Diabetic nephropathy class

According to implantation biopsies, the most frequent DN class was IIa (50% of cases), IIb was present in two cases, and classes 0, I and III in the others. At follow-up, 40% of cases remained stable in class IIa (patients 2,3,5 and 8), while two class IIb cases were downgraded to IIa and I (patients 1 and 6), respectively, and one class III case to IIb (patient 4). We observed a worsening in three cases: from class 0 to I (patient 7), from class I to IIb (patient 10) and from class IIa to IIb (patient 9) (Fig. 1). The GBM dimension was evaluated using TEM on follow-up biopsies and was available in seven patients. The thickness was 468 ± 311 nm in patient 1, 350 ± 50 nm in patient 2, 430 ± 80 nm in patient 4, 279 ± 65 nm in patient 6, 460 ± 50 nm in patient 7, 470 ± 50 in patient 8, 766 ± 100 in patient 10.

- (b) Interstitial fibrosis and tubular atrophy evaluation

Interstitial Fibrosis and tubular atrophy (IF/TA) scores showed not uniform changes. As shown in Table 3 each patient shows a different evolution, of note the three patients with shorter follow-up showed a worsening while the three with longer follow-up showed a decrease.

- (c) Vasculature evaluation

**Table 3:** The table depicts the histological scores of tubules and interstitium (from 0 to 3), arteriolar hyalinosis (from 0 to 2) and arteriosclerosis (from 0 to 2) of DN classification for the 10 patients enrolled in the study

Histopatological score		Patients									
		P1	P2	P3	P4	P5	P6	P7	P8	P9	P10
Tubules and interstitium	Pre	0	1	0	0	0	1	1	1	1	2
	FU	2	2	3	1	3	1	0	0	0	1
Arteriolar hyalinosis	Pre	1	2	1	2	2	2	1	2	2	0
	FU	0	0	1	2	1	1	2	1	2	2
Arteriosclerosis	Pre	1	2	–	1	1	–	2	1	2	1
	FU	1	2	–	2	1	2	1	1	0	1

‘Pre’ represents pre-implantation biopsy scores. ‘FU’ represents biopsy scores at follow-up visit

The DN arteriolar hyalinosis score improved in five patients, remained stable in three and worsened in two (Table 3). The arteriosclerosis could not be evaluated in one patient due to the sampling (patient 3), while five patients showed stable scores, one worsened, and the other improved (Table 3).

## Discussion

Diabetic donors’ potential to expand the donor pool has been extensively analyzed in large cohort studies; it has been ascertained that receiving a kidney from a DD does assure a survival benefit, compared to remaining on the waiting list and that patient and graft survival depend on the status of the recipient. Indeed, the worst outcomes are seen when diabetes is present in both the donor and the recipient, and the diabetic recipient of a non-diabetic kidney has a worse evolution then a non-diabetic recipient [3, 4, 10, 12, 13]. Although the differences in organ and patient survival rates between diabetic and non-diabetic donors are slight, the discard rate of DD is still too high and these organs are not utilized at their fullest potential [13, 14]. We present the histological and clinical analyses at long-term follow-up of a small but well-defined series of kidneys from ECD diabetic donors transplanted in non-diabetic elderly patients. Albeit the reversibility of diabetic lesions has already been established in the native kidneys of patients that received pancreas transplantation clear evidence is still lacking in the setting of kidney transplant from a diabetic donor to a non-diabetic recipient. In 1983, Abouna reported an almost complete resolution of the DN 7 months after kidney transplantation from a type 1 diabetic donor [5]; more recently, Harada showed a reversion of the DN lesions at the 1-year biopsy in 3 cases of kidney transplantation from living-DD to non-diabetic recipients [6], and Khan reported one case of DN regression [10]. Within our cohort of kidneys from DD, we selected a series of ten patients that had stayed non-diabetic during follow-up, and we evaluated their histology, graded the diabetes score at preimplantation biopsies and evaluated the evolution of the different lesions over time. The histological examination made at allocation and reviewed in light of the DN classification [7] evidenced the presence of DN glomerular lesions already at T0; they were mild in the majority of our patients, however, and only one presented class III. We did not identify a uniform trend in the DN lesions among the ten patients and at the different follow-ups. However, in 7 out 10 patients, DN lesions remained stable or downgraded during time, confirming the positive trend observed in the study of Harada and colleagues [7] whereas DN class worsened in 3 patients only. To the best of our knowledge, the evolution of DN after long-term euglycemia needs further evaluation. In fact, Truong et al. reported a histological evaluation of 11 diabetic donors, all with normal function and mild proteinuria. As in our own results, they found mild glomerular DN lesions in all cases at reperfusion biopsies, but one case with class III DN; however, no histology is available on recipients that were non-diabetic at follow-up [9, 15]. Our group has previously demonstrated that diabetic lesions encompassing mild and more severe classes of DN are already present in a clinical setting with still no signs of diabetic kidney disease (DKD) [16]. This discrepancy between clinic and pathological findings is increasingly reported in the literature, but it is not yet clear what determines these almost silent clinical and laboratory findings in the context of DN, potentially across all classes [9, 15, 17–20]. Since we showed that the evolution of DN glomerular lesions during normoglycemia can vary, more analyses and studies are needed to deepen our understanding of this intriguing evidence.

The degree of chronic injury by IF/TA was evaluated in allocation biopsies and the damage was substantially mild or absent, while arterio-nephrosclerosis was of mild-to-moderate entity in the same samples. Aside from DN, these lesions reflect the coexistence of hypertension, observed in 70% of our donors, and of high body weight or obesity, present in all of them. At follow-up, these lesions showed stability or mild histological progression, which could reflect the persistence of risk factors for vasculature damage over time: as mentioned, all our recipients were hypertense, with a high body weight and, in one case, severely obese. Truong’s diabetic recipients had a percentage of IF/TA analogue to ours, but the vascular damage was more severe and already evident in the post-reperfusion biopsies; the entity of the damages kept worsening, while arterial intimal fibrosis and arteriolar hyalinosis encompassed all the histological grades till the most severe, maybe because of a longstanding diabetes [9, 15].

In the context of kidney transplantation, chronic lesions of the tubulo-interstitium are the result of a number of processes that are still matter of debate; the ones identified in our patients were, for example, hypertension, high body weight/obesity, accelerated atherosclerosis, calcineurin inhibitors toxicity, evolution of ischemia reperfusion injury, ageing, polymorphisms in cytokine genes etc.[16, 21, 22]. IF/TA and interstitial inflammation are also part of DN classification and they have been linked to DKD clinical presentation. In fact, IF/TA is not present during the early stages and, once it appears, is considered responsible for the onset of albuminuria, due to albumin reabsorption failure [11, 17, 23, 24]. IF/TA in transplants can be the result of many factors but it is a known fact that diabetes contributes to its worsening; in our patients, euglycemia could perhaps ex-plain the slight IF/TA and vascular worsening.

The donors in our cohort had the highest number of years (54–77 y–o) and KDPI (80–100%) compared to the other published records. Singh et al. have recently reported 10-year graft and patient survival data analyzing the mis- matches in donor and recipient diabetes status; they reported a mean donor age of 36–51 years in the different groups, while the donors in the group DD- ND recipient had a mean age of 47 and a mean KPDI of 71% [4]. Similarly, Kahn describes a population of 38-year-old donors, 45-year old in the match DD-ND recipient [10]. In the large cohorts from OPTN, Mohan described a population of diabetic ECD donors with mean age 59.4 [12], while Cohen a diabetic population of donors with mean age 56 [13] and 51 years [3], with a mean KDPI of 81%. Finally, the histological data reported by Truong et al. referred to a population of donors with mean age 47.1 (18–72) [9, 15]. All the donors selected in our cohort matched the eligibility criteria to proceed with allocation, had normal renal function and proteinuria, when present, was mild. As emphasized by the KDPI, our population of donors was at very high risk for graft failure. This index, routinely used for allocation in the United States (US), calculates the risk of graft failure by comparing the donor to all kidney donors recovered the previous year and has demonstrated correlations with patient and graft outcomes [24], although limited to US donors, this index helps the assessment of donor risk profiles in European cohorts, too [25]. The recipients were as marginal and old as our donors. Again, they are the oldest among the cases reported in the literature. In Truong the age of recipients was 35–73 [9, 15]; recipients reported by Cohen were 55 (46–63) and 56 (47–64) [3, 13]; Khan described recipients with mean age 58 (49–66) years [10], the recipients reported by Singh were 54 (47–62) [4]; the age of the recipients reported by Mohan in the subgroup of diabetic-ECD donors is of 59.4 ± 6.2 years [12]. Even in marginal pairs like these, with very high-risk donors and old recipients, we did not observe a severe worsening at histology or in terms of GFR; they were both similar to the published data that refer to less comorbid donor-recipient couples [7, 9, 15]. We can hypothesize that the euglycemic milieu of non-diabetic recipients favors the stability or regression of the characteristic lesions of DN. Conversely, the worsening of kidney lesions we observed in 3 out 10 patients evaluated, can be referred to the presence of other concomitant comorbidities, namely hypertension and obesity. In fact, looking at individual data, those patients who had an impairment of DN lesions had an average BMI higher (29.7 kg/m^2^) than the remaining ones (23.5 kg/m^2^) and were treated with a greater number of blood pressure lowering drugs (3 as mean). Moreover, it has been well demonstrated that obesity and hypertension interact each other in triggering the worsening of kidney damage [26]. However, our study, given the observational design, can be interpreted as a hypothesis generating study, rather than a hypothesis testing. Moreover, our analysis is also limited by its small size and thus needs confirmation in large-scale prospective analyses. However, with the limitations of a case report study, it suggests that diabetic kidneys histologic lesions show variable evolution of its grading over time related to recipients characteristics such as euglycemic milieu (ameliorated lesions) or obesity and hypertension (worsening lesions).

## Data Availability

The datasets generated during and/or analysed during the current study are available from the corresponding author on reasonable request.
